# Micra Leadless and Transvenous Pacemaker: A Single‐Center Comparative Study of QRS Wave Duration Resulting From Different Pacing Sites

**DOI:** 10.1111/anec.70050

**Published:** 2025-01-27

**Authors:** Yichi Yu, Xiaomin Yang, Xiaoming Lian, Yan Zhao, Bo Liu, Xiangfei Feng, Qunshan Wang, Yigang Li

**Affiliations:** ^1^ Department of Cardiology, Xinhua Hospital, School of Medicine Shanghai Jiao Tong University Shanghai China

**Keywords:** Clinical: Electrophysiology—conduction disturbances, Clinical: Implantable devices—pacemaker‐bradyarrhythmias, Clinical: Implantable devices—physiologic pacing, Clinical: Non‐invasive techniques ‐ electrocardiography

## Abstract

**Objective:**

To compare the paced QRS duration on different sites in age‐, gender‐, and indication‐matched patients implanted with Micra leadless pacemakers and conventional transvenous pacemakers (TV‐PM).

**Method:**

A total of 82 patients from Xinhua Hospital, Shanghai Jiaotong University, were enrolled, including two groups of 41 patients matched according to gender, age, and pacemaker indications, who underwent Micra and TV‐PM implantations, respectively. The baseline data of the patients, the pacing site described using three‐ and nine‐partition methods, and the paced QRS duration on 12‐lead electrocardiogram were then analyzed.

**Results:**

Overall, patients in our population were on average 79.2 years of age and mostly male (75.6%). Atrioventricular node dysfunction was the most common indication (56.1%) for pacemaker therapy. Mid‐septum, especially Site 5, is the implantation site for most patients in both groups. Micra (145 ms) and TV‐PM (133 ms) both had the narrowest‐paced QRS at high septum, but Micra may exhibit significantly more reduced QRS duration than TV‐PM at low septum (Micra vs. TV‐PM: 143.0 [142.8–156.5] ms vs. 163.5 ± 17.5 ms, *p* = 0.044).

**Conclusion:**

The narrowest‐paced QRS complex for Micra leadless pacemakers is achieved at high septum, and pacing at low septum by Micra may acquire shorter QRS duration than conventional TV‐PM.

## Graphical abstract

The narrowest‐paced QRS complex for the Micra leadless pacemaker is observed at the high septum. Moreover, pacing at the low septum using the Micra may result in a shorter QRS duration compared to the conventional transvenous pacemaker.

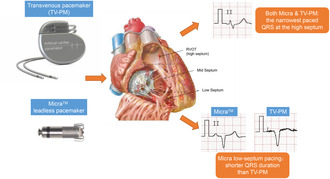

## 1 Introduction

Leadless pacemakers, like the Medtronic Micra transcatheter pacing system (TPS), present promising developments in terms of the simplicity and safety of implantation. This makes them an appealing alternative to traditional transvenous pacemakers (TV‐PMs), especially for patients who are at a higher risk of complications related to subcutaneous pouches or leads (Gi, Scacciavillani, and Narducci 2022; Reynolds et al. 2016). Although the Micra TPS does not support physiologic pacing modes, it has the distinct ability to immediately readjust the pacing site so as to obtain a satisfactory QRS duration on electrocardiograms (ECGs). This enables better biventricular synchrony and may potentially reduce adverse effects on long‐term cardiac function (Desai et al. 2006). The high ventricular septum, which can serve as a quasi‐physiologic pacing site close to the distal end of the His bundle or bundle branches (Curila et al. 2021), has been investigated for use in leadless pacemakers. However, its reliability is frequently questioned because of the lack of pectinate muscle support (Reddy et al. 2014). Consequently, debates regarding the optimal pacing site for leadless pacemakers continue.

Furthermore, the duration of the paced QRS wave can vary according to the implanted device. For example, the cathode‐to‐anode spacing in Micra is 18 mm (Omdahl et al. 2016; Lancellotti et al. 2019), while the tip‐to‐ring spacing of typical bipolar ventricular leads, such as the MRI Surescan 5076, is 10 mm (Lewicka‐Nowak et al. 2008). This difference in design might slightly change the ventricular activation vector during bipolar pacing, even when the pacing tips are placed in the same position. However, a direct comparison of the paced QRS width between Micra TPS and TV‐PM at the same pacing site has not been thoroughly investigated.

Hence, the present single‐center, retrospective, observational study endeavors to compare the paced QRS duration across different pacing sites in age‐, gender‐, and indication‐matched patients who have undergone implantation of either Micra TPS or TV‐PM.

## 2 Methods

### 2.1 Patient Population

**FIGURE 1:**
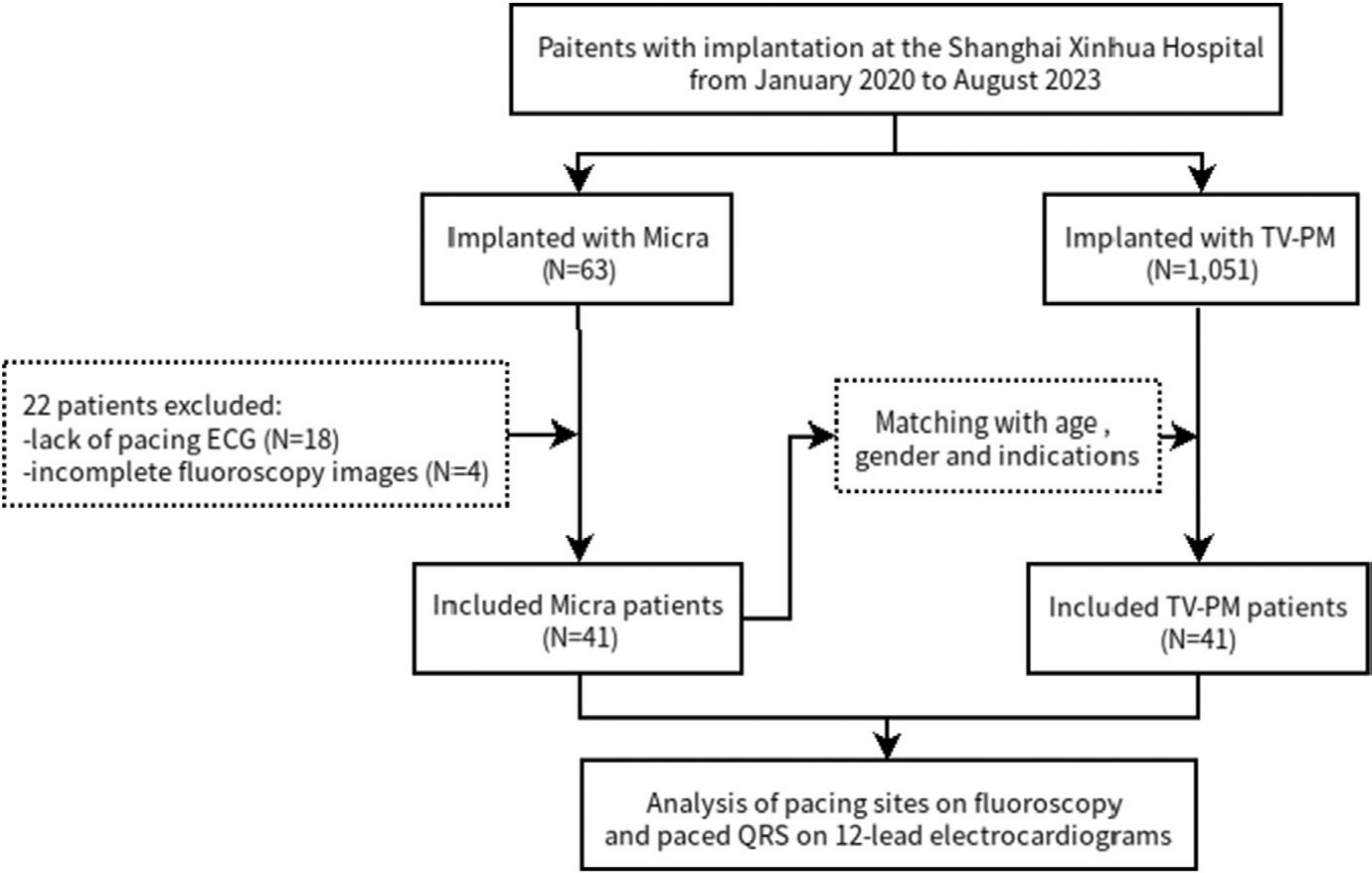
Workflow diagram of the study.

As detailed in Figure 1, we successively enrolled patients who met the guideline‐based indications for pacemaker implantation at Shanghai Xinhua Hospital, which is affiliated with the Faculty of Medicine, Shanghai Jiaotong University, from January 2020 to August 2023. During this time, a total of 63 patients had Micra TPS implants. To guarantee data integrity, patients with incomplete postoperative ECG or imaging data were excluded, resulting in a final study population of 41 Micra TPS patients.

To minimize the potential of selection bias associated with different procedures, we simultaneously screened 712 patients who had TV‐PM implantation within the same time period. Adhering to the same exclusion criteria, we selected 41 patients who were matched with the Micra TPS group in terms of age, gender, and procedural indications. It should be noted that patients receiving physiologic pacing (such as His bundle pacing or left bundle branch pacing) were excluded from both groups.

The indications for pacemaker implantation in this study were divided into two main groups: sinoatrial node (SAN) dysfunction, which includes sinus arrest, severe sinus bradycardia, sinoatrial block, and sick sinus syndrome; and atrioventricular node (AVN) dysfunction, which comprises high‐degree atrioventricular block and atrial fibrillation with bradycardia. This classification enabled a more in‐depth examination of the clinical characteristics and outcomes related to the two pacing systems.

Informed consent was obtained from all patients involved in this study. This ensured that each patient was fully aware of the nature, purpose, potential risks, and benefits of the implantation procedures (either Micra TPS or TV‐PM). They were provided with comprehensive information regarding the study, including how the data would be collected, stored, and used for analysis.

### 2.2 Procedure of Leadless and TV‐PM Implantation

All Micra TPS devices were implanted in strict accordance with the manufacturer's protocol (Ritter et al. 2015). Essentially, the small, leadless Micra pacemaker, connected to a delivery catheter, was inserted through the right femoral vein and guided to an exact location in the septum or apex of the right ventricle (RV). The choice of the implantation site was customized according to each patient's specific cardiac anatomy and clinical needs. Once the test parameters were confirmed to be satisfactory (R‐wave amplitude ≥ 5 mV, pacing threshold ≤ 1 V at 0.24 ms, and impedance in the range of 400–1500 Ω), the leadless pacemaker was released from the delivery system and firmly anchored to the right ventricular wall by four flexible nitinol tines. It is permitted for the operator to obtain a narrower QRS duration by fine‐tuning the position. However, it is necessary to prioritize ensuring the testing parameters and the stability of device, and fully consider the patient's tolerance level. The number of deployments in each Micra patient was recorded.

To ensure device stability, a pull test was carried out. During this test, the movement of the anchored tines was observed under high‐magnification visualization while gently pulling on the tether. Moreover, the optimal sensing and pacing thresholds were verified multiple times before cutting the tether. After the implantation was finished, an 8‐knot suture was applied to the local wound following the removal of the delivery catheter.

For patients receiving traditional TV‐PMs, the choice between single‐ or dual‐chamber pacing was not compulsory and was based on the individual patient's requirements. It should be noted that patients receiving physiologic pacing (such as His bundle pacing or left bundle branch pacing) were excluded, and the ventricular lead of all enrolled TV‐PM patients was MRI Surescan 5076. Similarly, the positioning of the ventricular lead was determined by the patient's specific anatomy and the results of the testing parameters.

### 2.3 Categorization of Pacing Site

Right ventricular imaging was carried out with a standard right anterior oblique (RAO) projection of 30° until the end of the left ventricular filling phase. Additionally, a left anterior oblique (LAO) projection of 45° was used to distinguish between septal and free‐wall positions. The postoperative analysis of the pacing site for each subject was conducted using the three‐ and nine‐partition methods, respectively, by two independent technicians.

**FIGURE 2:**
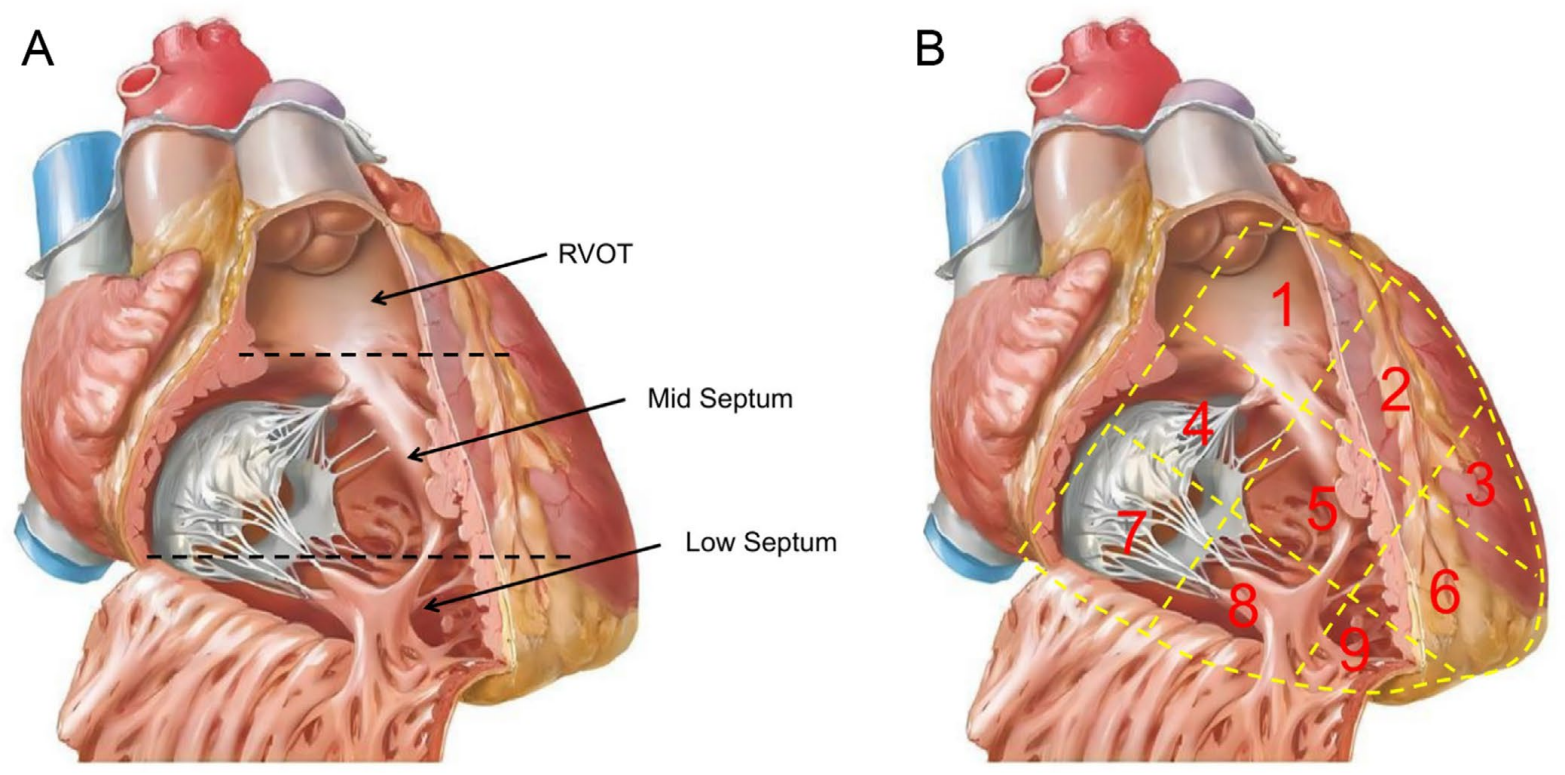
In RAO view, the RV is subdivided using three‐ (A) and nine‐partition (V) methods (B).

The three‐partition method (as illustrated in Figure 2A), as described by Kawakami et al. (2013), divides the RV into three regions on an RAO 30° view. The area above the supraventricular crest is defined as the right ventricular outflow tract (RVOT). The area from below the supraventricular crest to the level of the right ventricular anterior papillary muscle is considered the mid‐septal position. The area below the level of the right ventricular anterior papillary muscle is regarded as the low septum (apical position).

The nine‐partition method (as shown in Figure 2B), introduced by Su et al. (2020), was initially proposed for simplifying the localization of the left bundle branch pacing approach. In the RAO 30° view, the cardiac silhouette from the outer border of the heart to the left lateral border of the spinal column is divided into nine quadrants. To clearly describe the nine‐partition model, the 3 × 3 partitions are numbered from 1 to 9.

### 2.4 QRS Duration Under Ventricle Pacing

All the enrolled patients underwent a standardized postoperative resting 12‐lead ECG with a paper speed of 25 mm/s and a sensitivity of 10 mm/mV. A persistent VVI pacing mode was set at 90 beats per minute (bpm) to rule out the possibility of fusion beats. For the analysis of the QRS wave, two independent technicians, who were unaware of the type of pacemaker and pacing site, carried out manual assessments. The QRS duration measurements were accurately obtained using the open‐source imaging platform, Weasis Medical Viewer version 3.8, which ensured accuracy and reproducibility.

### 2.5 Statistical Analysis

All statistical analyses were conducted utilizing SPSS version 25.0 (IBM statistics). The Kolmogorov–Smirnov test was employed to examine the normal distribution of continuous variables. Data are presented as mean ± standard deviation (parametric) or median ± 25th and 75th percentiles (nonparametric). Comparisons between continuous variables were carried out using the Student's *t*‐test (parametric) or the Kruskal–Wallis test (nonparametric). Categorical variables are presented as numbers and percentages and compared by means of the Chi‐squared test. A *p*‐value less than 0.05 was considered statistically significant.

## 3 Results

### 3.1 Baseline Characteristics

As presented in Table 1, a total of 82 patients were enlisted, with 41 patients having the Micra leadless pacemaker implanted and 41 patients with TV‐PM, matched in terms of age, gender, and indications. The patients had an average age of 79.2 ± 8.0 years and were predominantly male (75.6%). AVN dysfunction constituted the majority of the indications for pacemaker therapy in our study population (56.1%). Regarding comorbidities, there was no significant difference in high blood pressure, Type 2 diabetes, atrial fibrillation, and renal dysfunction. However, there was a tendency toward a higher proportion of coronary artery disease in patients with Micra (46.3% vs. 24.4%, *p* = 0.064). The only characteristic that showed a significant difference was the left ventricular ejection fraction (LVEF%), which had a slightly higher value in Micra patients compared to TV‐PM (65.8 ± 7.7 vs. 61.3 ± 6.0, *p* = 0.003).

**TABLE 1:** Baseline characteristics of Micra leadless and TV‐PM patients.

	Overall (*n* = 82)	Micra leadless (*n* = 41)	TV‐PM (*n* = 41)	*p*
Age	79.2 ± 8.0	79.3 ± 8.1	79.1 ± 8.0	0.913
Gender: female	20 (24.4%)	10 (24.4%)	10 (24.4%)	1.000
Indication for implantation				
SAN dysfunction	36 (43.9%)	18 (43.9%)	18 (43.9%)	1.000
AVN dysfunction	46 (56.1%)	23 (56.1%)	23 (56.1%)	1.000
Hypertension	57 (69.5%)	28 (68.3%)	29 (70.7%)	1.000
Type 2 diabetes	19 (23.2%)	11 (26.8%)	8 (19.5%)	0.601
Coronary artery disease	29 (35.4%)	19 (46.3%)	10 (24.4%)	0.064*
Atrial fibrillation	26 (34.1%)	13 (34.1%)	13 (34.1%)	1.000
Creatinine (μg/mL min)	78 (68.1–101.7)	79 (68–112)	78 (69–99.1)	0.697
LVEF%	63.6 ± 7.0	65.8 ± 7.7	61.3 ± 6.0	0.003**

*Note:* Continuous data were presented as mean ± SD (parametric) or median ± 25th and 75th percentile (nonparametric); categorical as number (%).

Abbreviations: AVN, atrioventricular node; LVEF%, left ventricular ejection fraction; SAN, sinoatrial node; TV‐PM, transvenous pacemaker.

* *p* < 0.1.

** *p* < 0.01.

**FIGURE 3:**
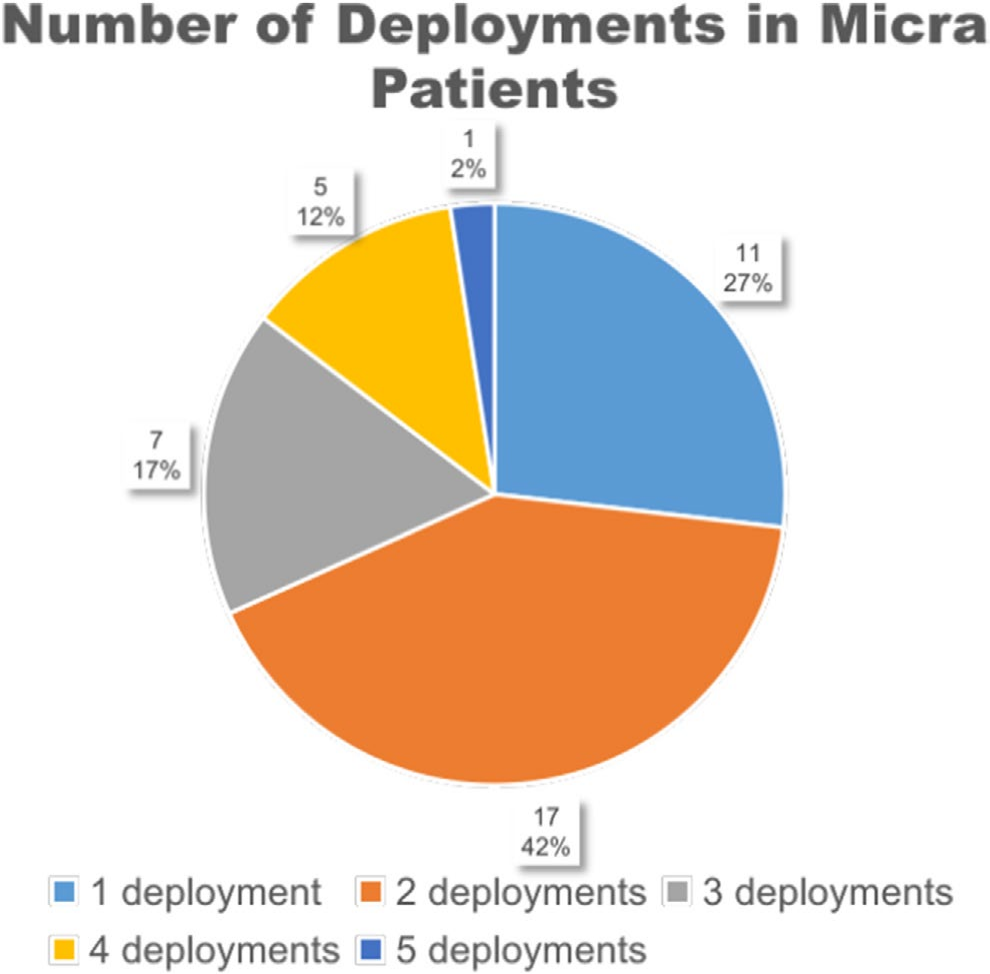
Number of deployments in Micra patients.

As illustrated in Figure 3, the number of device deployment attempts for Micra patients was retrospectively analyzed. All Micra patients underwent a minimum of one and a maximum of five deployment attempts (with an average of 2.25 attempts). Among them, 27% of the patients achieved successful deployment in one attempt, while the proportions of patients with multiple successful deployments were 42% (two deployments), 17% (three deployments), 12% (four deployments), and 2% (five deployments) respectively.

### 3.2 Device or Lead Position

A significant difference in device or lead position was demonstrated between the two groups (as detailed in Table 2). According to right ventriculography, the mid‐septum was the preferred position for the majority of both Micra (63.4%) and TV‐PM (51.2%) patients in our study population. Nevertheless, a lower septum was selected as the deployment target for more TV‐PM (46.3%) patients compared to Micra (22.0%) patients. In contrast, more Micra (14.6%) patients had deployment in the RVOT than TV‐PM (2.4%) patients.

**TABLE 2:** Comparison of paced QRS duration between Micra and TV‐PM at different pacing sites.

	Micra (*n* = 41)		TV‐PM (*n* = 41)		*p*
	Patient number	QRS duration (ms)	Patient number	QRS duration (ms)	
Three‐partition method					
High Septum (RVOT)	6 (14.6%)	145.4 ± 12.2	1 (2.4%)	133	—
Mid‐Septum	26 (63.4%)	149.6 ± 11.3	21 (51.2%)	144.0 (140.2–151.0)	0.285
Low Septum	9 (22.0%)	143.0 (142.8–156.5)	19 (46.3%)	163.5 ± 17.5	0.044**
Nine‐partition method					
Site 1	0 (0%)	—	0 (0%)	—	—
Site 2	5 (12.2%)	147.4 ± 12.5	1 (2.4%)	133	—
Site 3	1 (2.4%)	135	0 (0%)	—	—
Site 4	1 (2.4%)	141	0 (0%)	—	—
Site 5	21 (51.2%)	150.4 ± 12.9	16 (39%)	143.9 (141.2–150.1)	0.27
Site 6	6 (14.6%)	150.2 ± 9.3	6 (14.6%)	151.5 ± 25.2	0.907
Site 7	0 (0%)	—	4 (9.8%)	152.2 ± 4.0	—
Site 8	7 (17.1%)	150.4 ± 13.6	10 (24.4%)	163.5 ± 16.9	0.097*
Site 9	0 (0%)	—	4 (9.8%)	179.4 ± 19.5	—

*Note:* Continuous data were presented as mean ± SD (parametric) or median ± 25th and 75th percentiles (nonparametric); categorical as number (%).

Abbreviation: RVOT, right ventricular outflow tract.

* *p* < 0.1.

** *p* < 0.05.

Under the nine‐partition method, the positional differences between the two groups were further elaborated (Table 2). Among the six Micra patients with RVOT implantation, five were implanted at Site 2 and one at Site 3. The sole TV‐PM patient with RVOT deployment was at Site 2. For patients with mid‐septal implantation, both groups had the highest number of patients deployed at Site 5 (21 patients in the Micra group and 16 patients in the TV‐PM group). Six patients were implanted at Site 6 under each of the two different implantation devices. For patients implanted at the low septum, all Micra patients were implanted at Site 8. However, among TV‐PM patients, four were implanted at Site 7, 10 patients at Site 8, and four patients at Site 9.

### 3.3 Paced QRS Width

**FIGURE 4:**
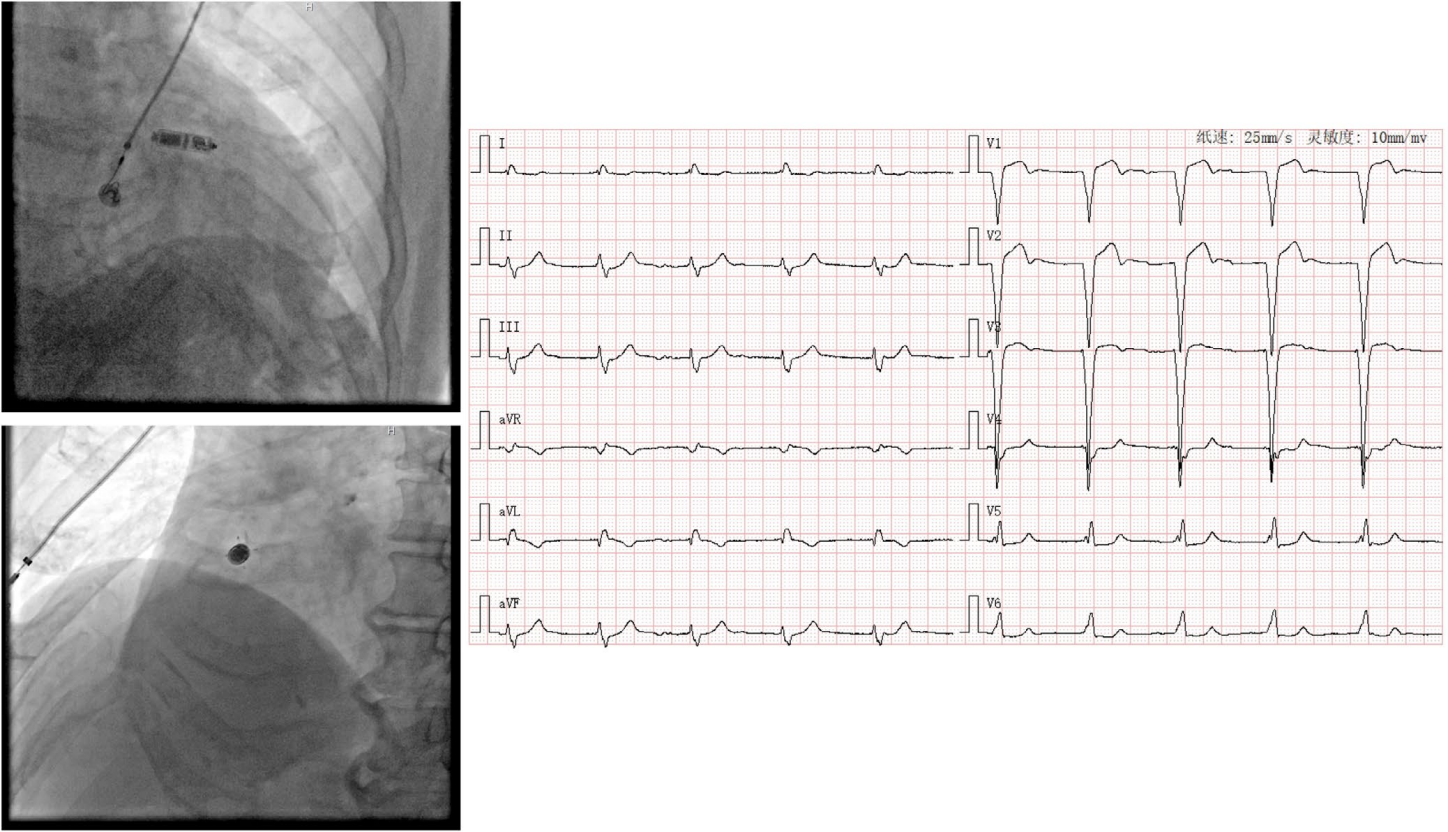
Representative case of Micra implantation at RVOT (Site 3) with paced QRS wave duration of 150 ms and RS pattern in Leads II, III, and aVF.

**FIGURE 5:**
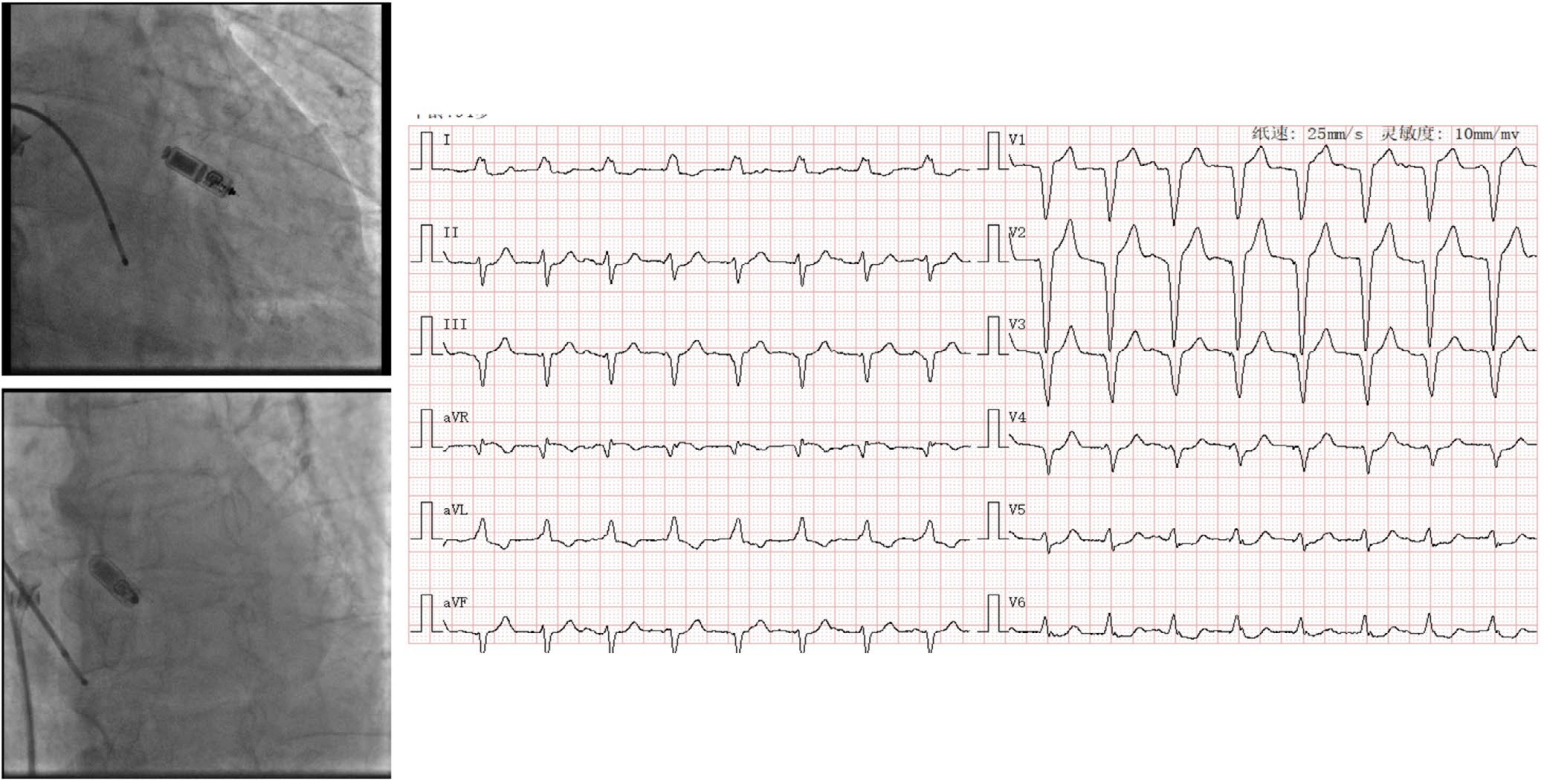
Representative case of Micra implantation at mid‐septum (Site 5) with paced QRS wave duration of 140 ms and LBBB (left bundle branch block) pattern and RS pattern in Leads II and aVF. QRS transition in Lead V5.

**FIGURE 6:**
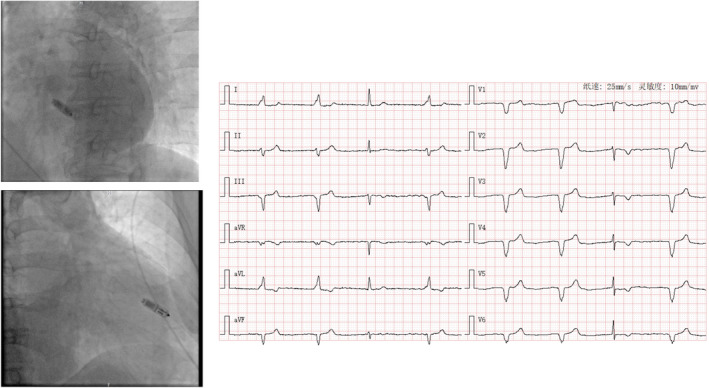
Representative case of Micra implantation at mid‐septum (Site 6) with paced QRS wave duration of 166 ms. Paced QRS morphology is characterized as negative R in Lead III, aVF, and V4–V6 and RS pattern in Lead II. No QRS transition is noted in precordial leads.

**FIGURE 7:**
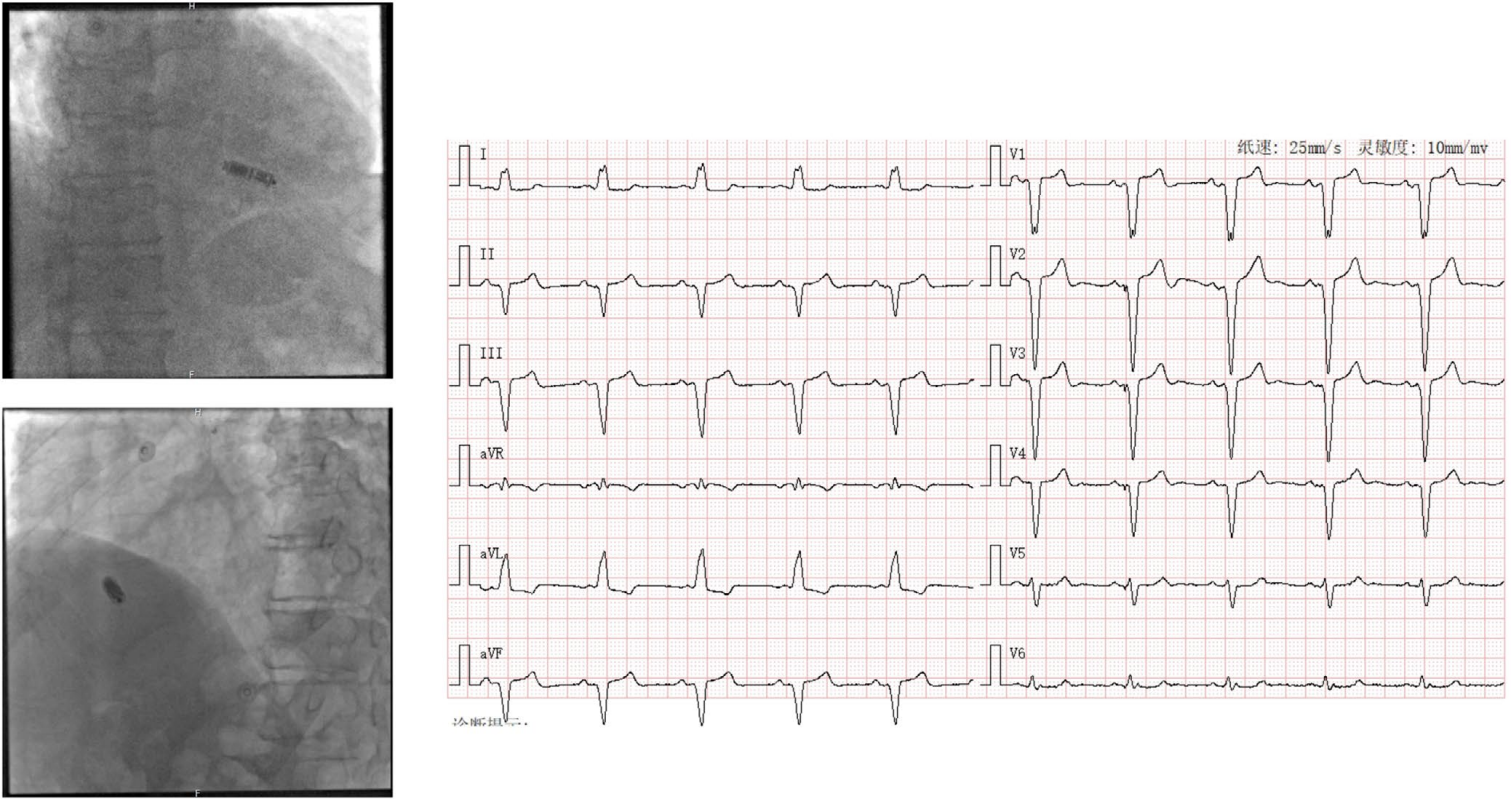
Representative case of Micra implantation at low septum (Site 8) with paced QRS wave duration of 143 ms and LBBB (left bundle branch block) pattern and negative R waves in Leads II, III, and aVF. QRS transition in Lead V5.

**FIGURE 8:**
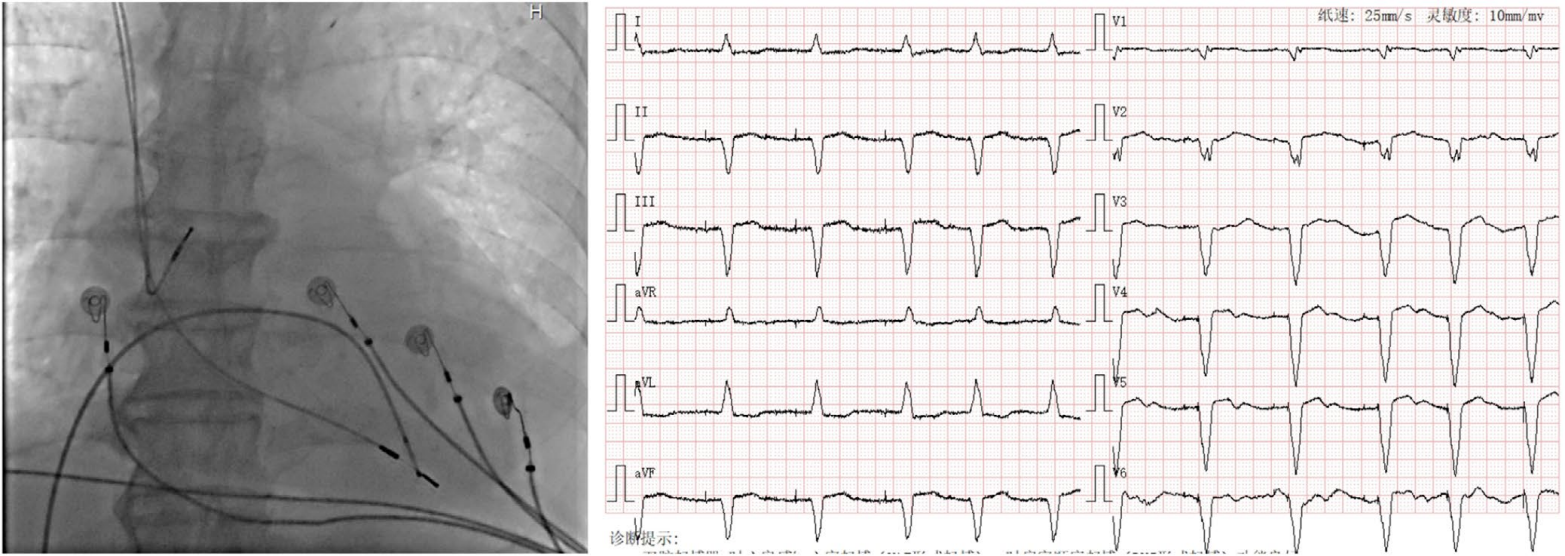
Representative case of TV‐PM implantation at low septum (Site 9). Wide‐paced QRS wave with duration of 185 ms as shown in EKG.

The characteristics of paced QRS duration on ECG in relation to the Micra device and TV‐PM lead implant location are presented in Table 2. Firstly, wider‐paced QRS complexes were observed at the low‐septal position compared to the RVOT or mid‐septum, especially in TV‐PM patients. However, this difference in QRS duration was not equally evident in patients implanted with the Micra device. Secondly, there was no significant difference in QRS duration between the two groups of patients implanted at the mid‐septum [Micra vs. TV‐PM: 149.6 ± 11.3 ms vs. 144 (140.2–151.0) ms, *p* = 0.285]. When divided by the nine‐partition method, there was also no significant difference either at Site 5 [Micra vs. TV‐PM: 150.4 ± 12.9 ms vs. 143.9 (141.2–150.1) ms, *p* = 0.270] or Site 6 (Micra vs. TV‐PM: 150.2 ± 9.3 ms vs. 151.5 ± 25.2 ms, *p* = 0.907). However, an intriguing finding was that the QRS complex width at the low septum in the Micra group was significantly narrower than that in the TV‐PM group (Micra vs. TV‐PM: 143.0 [142.8–156.5] ms vs. 163.5 ± 17.5 ms, *p* = 0.044). Even after eliminating implant locations at Sites 7 and 9 using the nine‐partition method, a trend of narrower QRS in the Micra group compared to the TV‐PM group was still noticed under implantation at Site 8 (Micra vs. TV‐PM: 150.4 ± 13.6 ms vs. 163.5 ± 16.9 ms, *p* = 0.097). (Representative cases of Micra and TV‐PM implantation are shown from Figures 4, 5, 6, 7, 8).

## 4 Discussion

This study is the first of its kind to date to horizontally compare the paced QRS wave duration between the Micra leadless pacemaker and the conventional TV‐PM in a real‐world population. Although the narrowest QRS complex was observed at the high septum with both methods, interestingly, we have found that pacing at the low septum using the Micra may result in a relatively narrower duration compared to TV‐PM.

Originally, the RV apex was regarded as the preferred implantation site for both the Micra TPS and TV‐PM because of the ease of the procedure. However, pacing from the apex stimulates myocardial cells rather than Purkinje fibers, which broadens the paced QRS duration. Permanent RV apex pacing may have harmful effects on ventricular remodeling and increase the risks of adverse cardiovascular events (Simantirakis et al. 2020; Burri et al. 2011). Therefore, in recent studies, RVOT or mid‐septal pacing has been considered the preferred pacing site due to the narrower‐paced QRS (Sharma et al. 2020; Yuan et al. 2023), and this view has naturally been extended to the implantation of leadless pacemakers.

Firstly, our results confirm the advantages of high‐septal pacing. As reported in previous studies (Sharma et al. 2020; Yuan et al. 2023), compared to mid‐septum and apex pacing, high ventricular septal pacing produces a relatively narrower QRS wave with nearly synchronous biventricular contraction because of selective or nonselective His bundle or bundle branch capture. Given the firmness of the ventricular lead, we prefer to use special spiral electrodes, such as the Medtronic 3830 lead, on the high septum and simultaneously achieve physiological pacing. Although the extension of the pectinate muscle to the RVOT may be seen through right ventriculography (Yuan et al. 2023), the feasibility of choosing an implantation point remains unclear.

**FIGURE 9:**
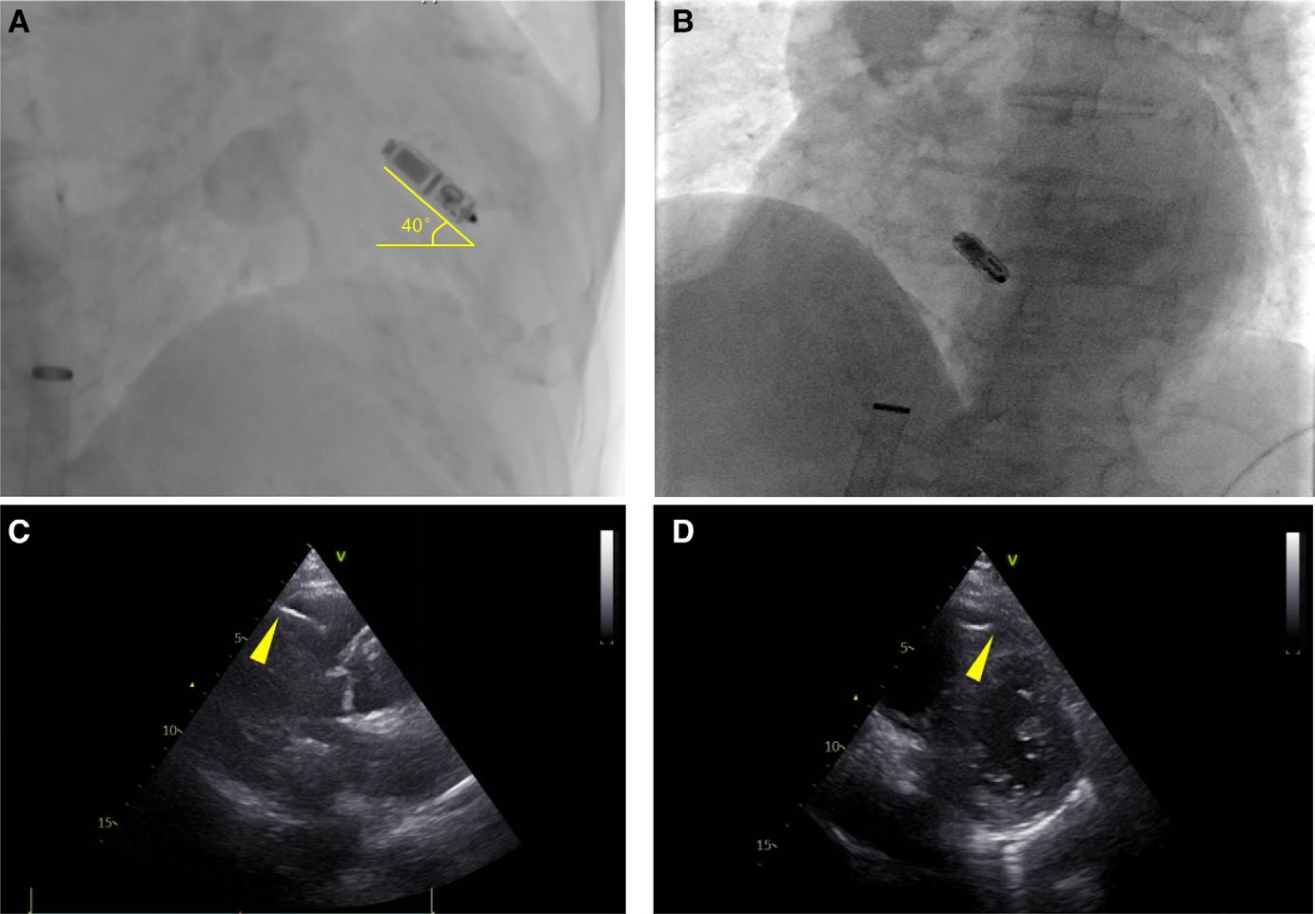
(A, B) Representative image of anterior mid‐septal Micra implantation, in which a high angle of approximatively 40° (marked in yellow) between Micra and horizontal line is illustrated in RAO view. (C, D) As shown in echocardiography, the anode of Micra was tightly screwed to RV anterior mid‐septum (yellow arrows).

Secondly, our findings re‐evaluate the low septum as a noninferior implantation site for the Micra. The RV low septum and apex are rich in pectinate muscle (Shenthar et al. 2020) and were initially recommended as hooking locations for the Micra. Despite having a more asynchronous depolarization sequence than the high septum, a narrower QRS wave than TV‐PM may still be achieved at the lower site. We ascribe this new finding to the following hypotheses: (i) The Micra has a larger cathode‐to‐anode spacing than the conventional ventricular lead, which permits a wider range of depolarization loop; (ii) a different depolarization vector due to the higher angle of direction of the Micra (a representative case in our population as shown in Figure 9); and (iii) opportunistic capture of other unique anatomic structures (e.g., moderator band) where conduction tissue extends (Jiang et al. 2020). Although more evidence and research are still required, through this study, we still recognize that low‐septal pacing with the Micra can simultaneously balance biventricular synchrony and implantation safety.

During the postprocedural follow‐up period, the Micra group generally had a fewer number of patients affected by complications (Table 3). In the Micra group, among the 41 patients, none had pericardial effusion, dislodgement, or device‐related infections. Only one patient each had diaphragmatic pacing and groin hematoma, and no patient had pneumothorax. In contrast, for the TV‐PM group, while no patient had pericardial effusion, two patients had dislodgement, one had a device‐related infection, one had diaphragmatic pacing, two had groin or pocket hematoma, and two had pneumothorax.

**TABLE 3:** Postprocedural complications.

	Micra (*n* = 41)	TV‐PM (*n* = 41)
Pericardial Effusion	0	0
Dislodgement	0	2
Device‐related Infections	0	1
Diaphragmatic pacing	1	1
Groin or Pocket Hematoma	1	2
Pneumothorax	0	2

In conclusion, based on the analysis in this paper of paced QRS by the Micra TPS and conventional TV‐PM, we believe that there is no complete similarity in terms of QRS duration at the same pacing site. When the Micra has to be implanted at the low septum under various limitations, it is recommended to refer to the actual QRS wave duration under pacing for comprehensive assessment.

### 4.1 Study Limitations

We present a nonrandomized retrospective monocentric study with only 82 patients, which may limit the generalizability of our conclusion. Although the low septum has been demonstrated to be a safe pacing site for both leadless and TV‐PMs, the underlying mechanism for the narrower QRS duration in Micra patients still requires further studies for clarification. Additionally, long‐term follow‐up for variations in QRS duration and prognosis may be necessary in our future research.

## 5 Conclusion

The narrowest‐paced QRS complex for the Micra leadless pacemaker is observed at the high septum. Moreover, pacing at the low septum using the Micra may result in a shorter QRS duration compared to the conventional TV‐PM.

## Author Contributions

The authors take full responsibility for this article.

## Conflicts of Interest

The authors declare no conflicts of interest.

## Data Availability

The authors have nothing to report.
